# Supplementary figures and images for: Formalin-induced pain prolongs sub- to supra-second time estimation in rats
Source: PeerJ. 2021 Mar 2;9:e11002. doi: 10.7717/peerj.11002 (PMC7934679; doi:10.7717/peerj.11002)

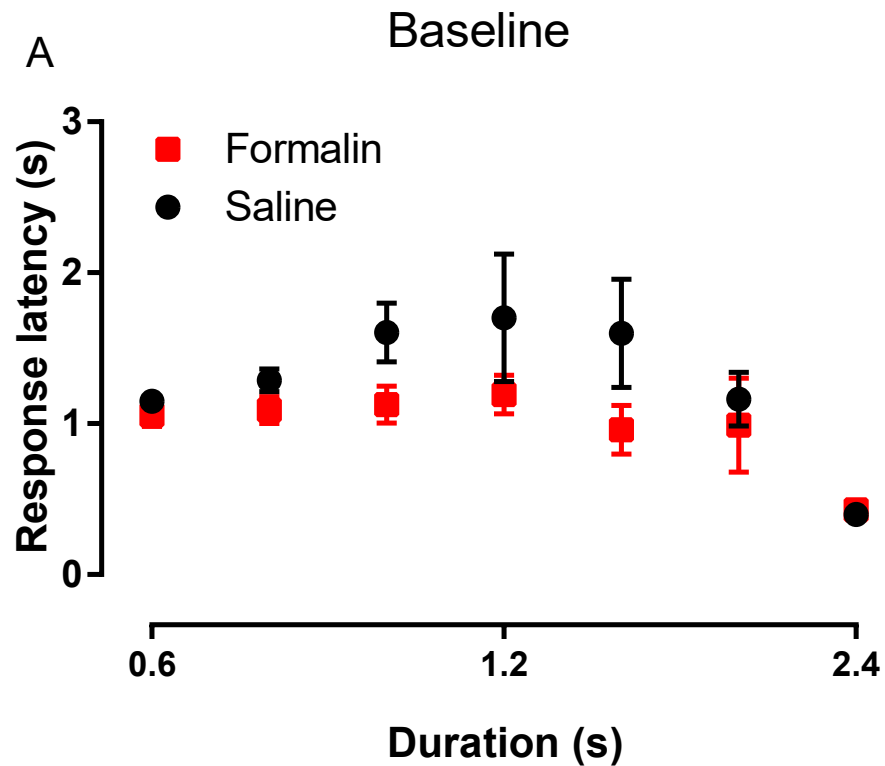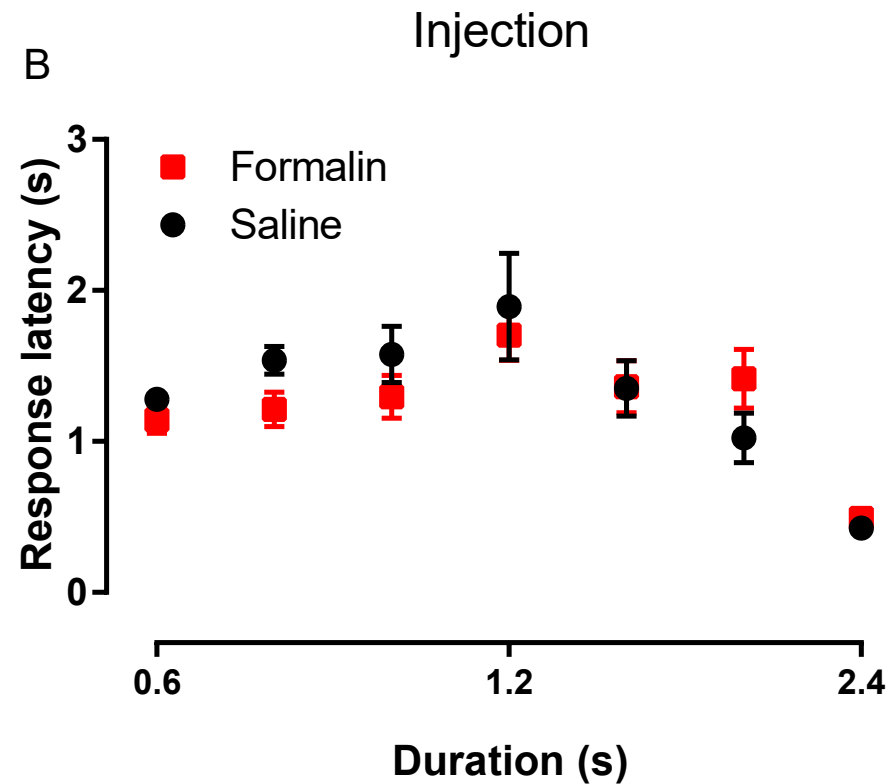

Supplement: Figure S1 — There is no significant difference between formalin group and saline group in each session. [file peerj-09-11002-s003.pdf]

## Baseline

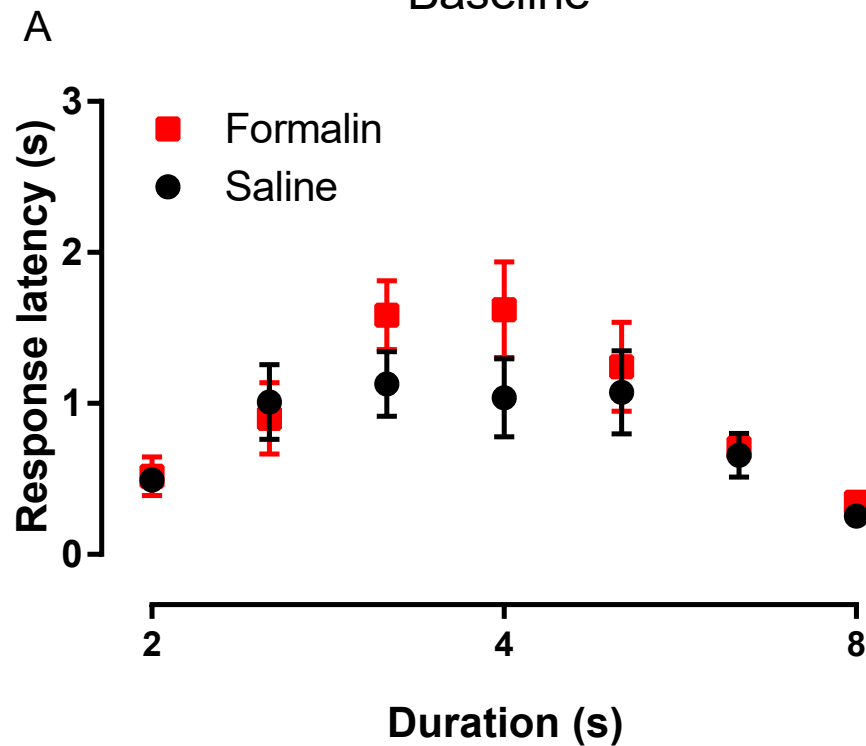

## Injection

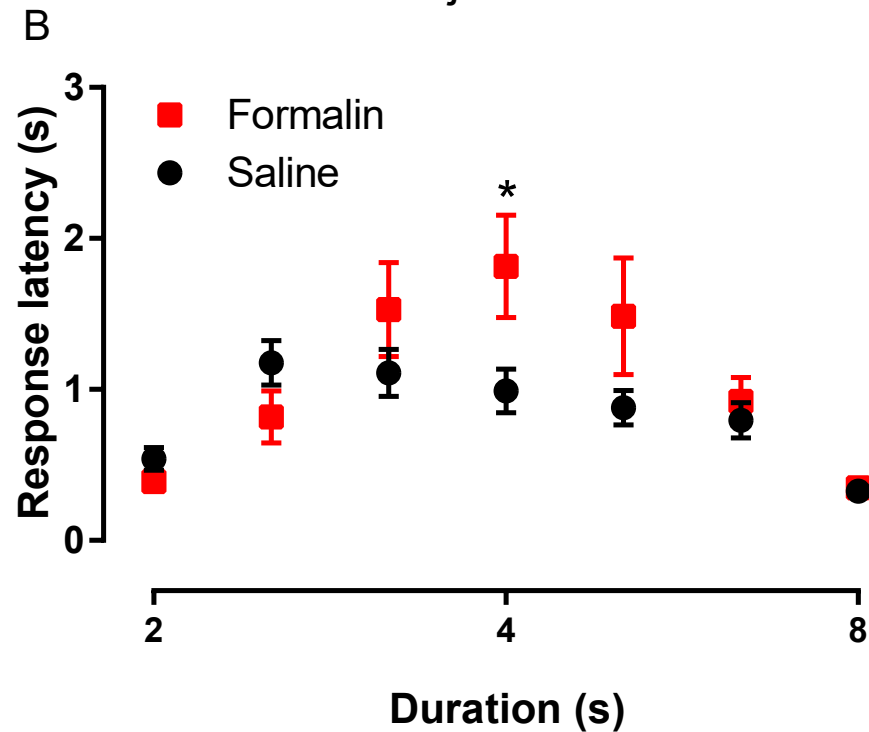

Supplement: Figure S2 — Formalin treatment prolonged response latency to 4-s trials and 5.04-s trials in the injection test (B). *p < 0.05, **p < 0.01 vs. saline group. [file peerj-09-11002-s004.pdf]

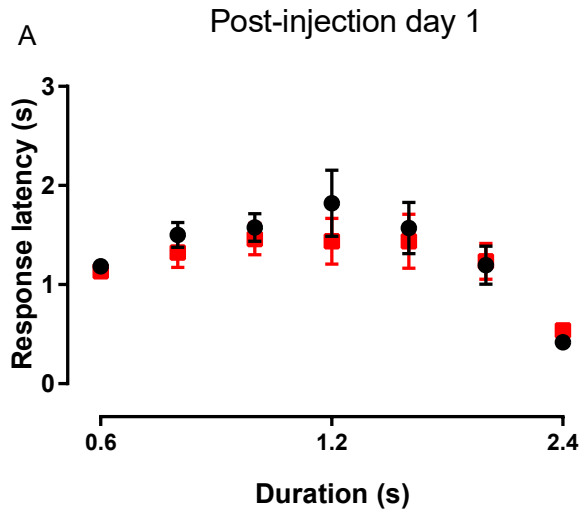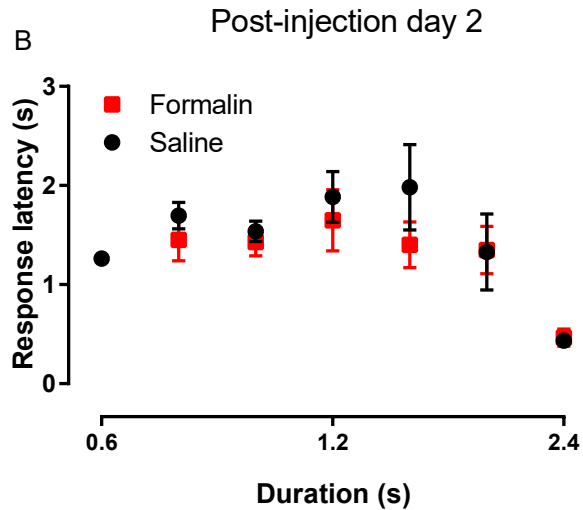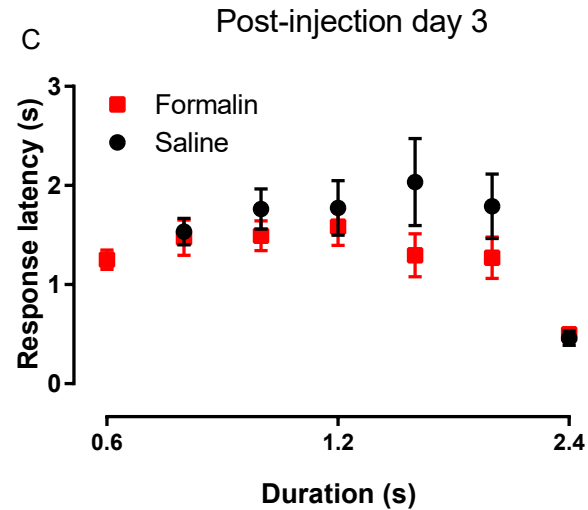

Supplement: Figure S3 — There is no significant difference between formalin group and saline in each session. [file peerj-09-11002-s005.pdf]

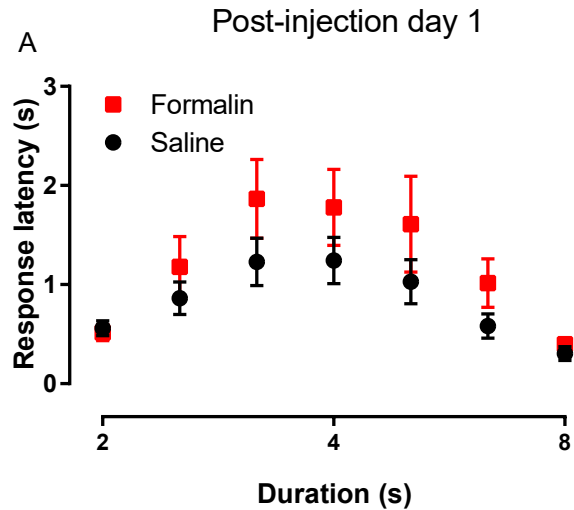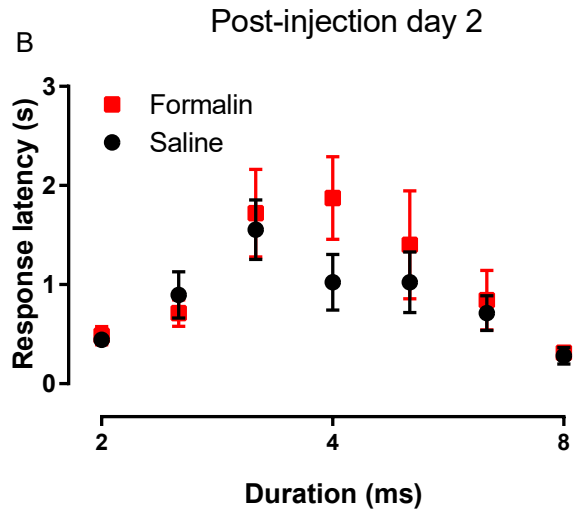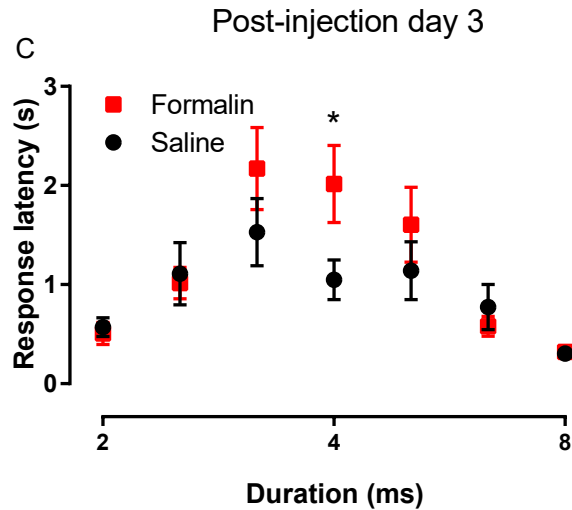

Supplement: Figure S4 — Formalin treatment prolonged the response latency to 4-s trials in post-injection day3 (C). *p < 0.05 vs. saline group. [file peerj-09-11002-s006.pdf]
